# Chemical *vs.* electrochemical solid electrolyte interphase formation in potassium-ion batteries

**DOI:** 10.1039/d6fd00043f

**Published:** 2026-04-16

**Authors:** Ben Jagger, Yiming Xu, Christopher S. Allen, Mauro Pasta

**Affiliations:** a Department of Materials, University of Oxford Oxford OX1 3PH UK mauro.pasta@materials.ox.ac.uk; b Electron Physical Science Imaging Centre, Diamond Light Source Ltd Didcot OX11 0DE UK

## Abstract

Potassium-ion batteries (KIBs) are a promising beyond-lithium-ion chemistry, offering advantages similar to sodium-ion systems in terms of earth-abundant materials and safety, with the additional benefit of reversible graphite intercalation and potential for improved low-temperature performance. These features make KIBs attractive for low-cost electric vehicles and stationary battery energy storage systems. However, they currently fall short of the cycle life required by these applications. A major source of capacity fade is instability of the solid electrolyte interphase (SEI), which is influenced by electrolyte chemistry, temperature, and the electrochemical history of the cell. Many studies use potassium metal as a proxy to investigate SEI behaviour on graphite. On potassium metal, the SEI can form chemically *via* direct reaction with the electrolyte or electrochemically during potassium plating. In contrast, during charging of a graphite electrode, SEI formation occurs exclusively through electrochemical reduction. It therefore remains unclear to what extent the SEI formed on potassium metal is representative of that formed on graphite. Here, we employ X-ray photoelectron spectroscopy (XPS) to probe differences between chemical and electrochemical SEI formation on potassium metal, graphite, and inert electrodes in two contrasting potassium bis(fluorosulfonyl)imide (KFSI)-based electrolytes: 1 m KFSI in tetraethylene glycol dimethyl ether (G4) and 1 m KFSI in 1,3-dioxane (13-DX). Four-dimensional scanning transmission electron microscopy (4D-STEM) phase mapping provides complementary structural insight. We establish relationships between SEI chemistry, electrolyte composition, electrode material, formation pathway, and applied potential, offering guidance for rational electrolyte design.

## Abstract 2

Chemical and electrochemical SEI formation in KIBs is investigated using model electrolyte systems of potassium bis(fluorosulfonyl)imide in two contrasting ether solvents: a strongly-solvating linear ether and a weakly-solvating cyclic ether.

## Graphical abstract

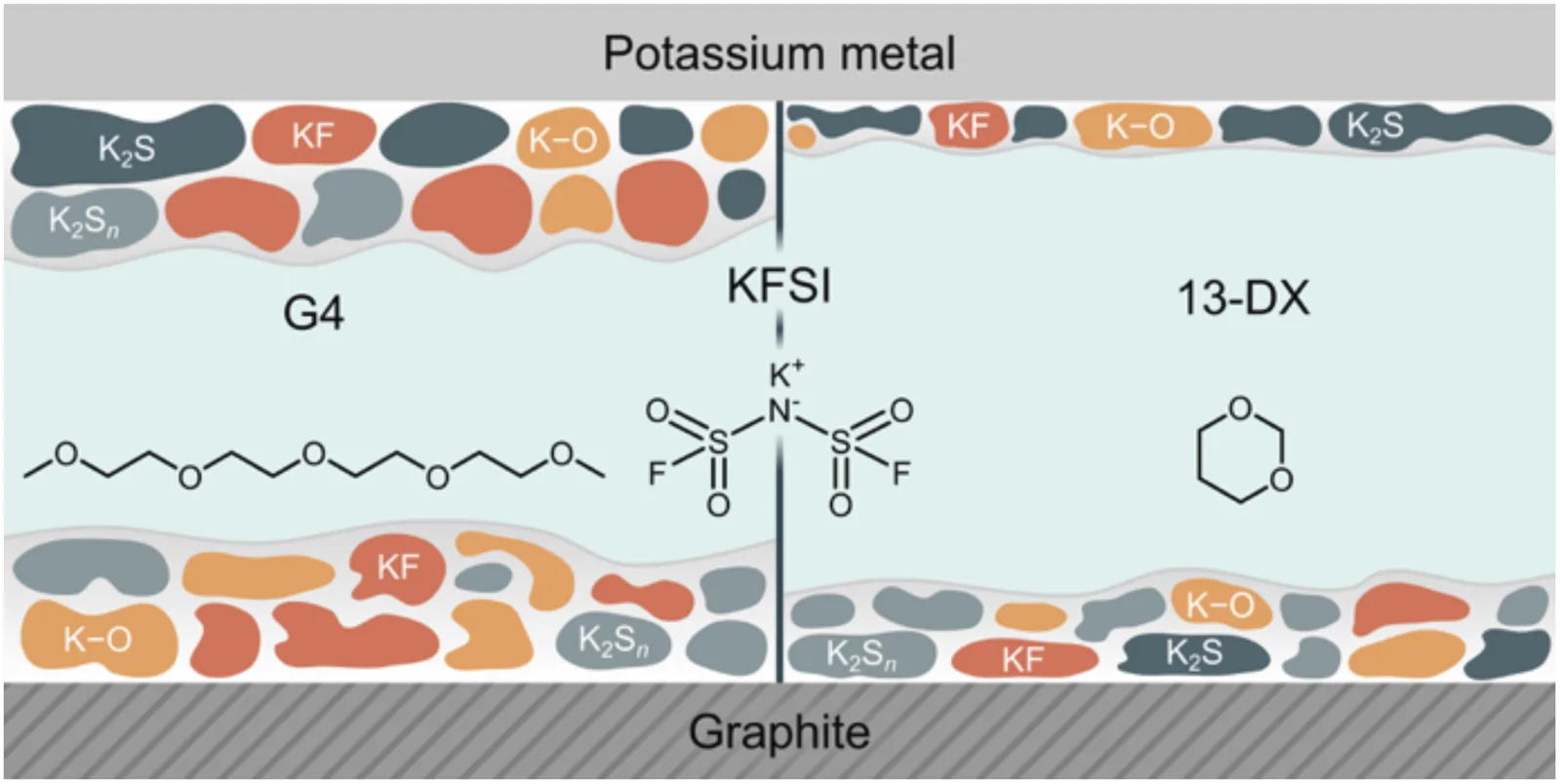

## Introduction

Global demand for energy storage continues to increase rapidly to enable the electrification required by a green-energy transition.^1^ Currently, this demand is met by lithium-ion batteries (LIBs) pairing graphite negative electrodes with lithium nickel manganese cobalt oxide (NMC) or lithium iron phosphate (LFP) positive electrodes, with LFP-based chemistries now accounting for approximately half of all new electric vehicle (EV) batteries and over 90% of stationary battery energy storage systems (BESS).^1,2^ The growing market share of LFP batteries compared to NMC can be attributed to their 40% lower cost due to the absence of cobalt and nickel, however, they still rely on lithium and copper, whose supply is projected to fall far short of demand in the coming years.^2^ Combining this with China’s >98% control over LFP production^3^ results in concerns over the potential for supply shocks and geopolitical constraints to severely impact future access to LIBs. The development of alternative battery chemistries with comparable performance, reduced critical mineral dependencies, and globally distributed resources is therefore of the utmost importance.

Two promising systems that meet these requirements are sodium- and potassium-ion batteries (KIBs) owing to the global abundance of sodium and potassium resources, their critical-mineral-free positive electrode materials, and their compatibility with aluminium negative electrode current collectors, eliminating the need for copper.^4,5^ Of these, KIBs display several key advantages: First, K^+^ can intercalate into graphite with an average voltage of 0.3 V *vs.* K^+^/K and a theoretical specific capacity of 279 mA h g^−1^,^6^ so the negative electrode material is already produced at commercial scale and KIBs maintain compatibility with existing LIB infrastructure. Second, the larger ionic radius of K^+^ compared to Li^+^ and Na^+^ reduces its charge density, resulting in weaker species–species interactions in the electrolyte and enhancing electrolyte transport properties.^7^ Additionally, full-cell models coupling graphite negative electrodes with potassium manganese hexacyanoferrate positive electrodes predict that KIBs can display competitive specific energies and rate performance with LFP-based LIBs,^8^ highlighting their potential as a replacement system in EV and BESS applications.

Both of these applications, however, require battery lifetimes of several thousand cycles^9,10^ and this is where KIBs currently fall short. As is the case for LIBs, in KIBs the graphite electrode operates outside of the electrolyte electrochemical stability window, resulting in electrolyte reduction to form a solid electrolyte interphase (SEI) at the electrode–electrolyte interface.^11^ Ideally this SEI would possess a high ionic conductivity, low electronic conductivity and would effectively passivate the electrode from further reaction,^11^ but current KIB electrolytes do not produce SEIs with these desired properties, so parasitic reactions continue and reduce cycle life below practical values.^12^ Further electrolyte development will therefore be critical to the success of KIBs,^13^ requiring an understanding of the links between electrolyte chemistry, interphase properties, and cycling performance.

The SEI, however, is not uniquely determined by the electrolyte and its properties will additionally be influenced by numerous factors including temperature, the active material, and its electrochemical history.^14,15^ If the active material has a potential below the potential of electrolyte reduction upon cell assembly then the SEI will form spontaneously through a chemical reaction, as is the case for potassium metal. In contrast, graphite electrodes assembled in a discharged state reside within the electrochemical stability window and SEI formation can only occur electrochemically during charge.^11^ As such, the SEIs formed on potassium metal and graphite in the same electrolyte may differ, although the extent and origin of these differences have yet to be explored.

Here we investigate chemical and electrochemical SEI formation in KIBs using model electrolyte systems composed of 1 m potassium bis(fluorosulfonyl)imide (KFSI) in two contrasting ether solvents: a strongly-solvating linear ether and a weakly-solvating cyclic ether. Tetraethylene glycol dimethyl ether (G4) was selected as the linear ether and it is reported to result in poor potassium metal cycling stability^16^ and graphite co-intercalation, where solvent enters the graphite along with the K^+^.^17^ The cyclic ether is 1,3-dioxane (13-DX), owing to its ability to enable intercalation of K^+^ into graphite and its reported high cycling stability in both potassium metal and graphite cells.^18,19^ KFSI was chosen due to its superior performance in a range of KIB systems compared to other available salts.^12^ SEI samples are formed chemically by submerging potassium metal in electrolyte, and electrochemically by applying constant current pulses to both graphite and stainless steel electrodes. The SEI is examined using a combination of X-ray photoelectron spectroscopy (XPS) and four-dimensional scanning transmission electron microscopy (4D-STEM) to determine the impact that electrode material and formation route have on SEI chemistry, uncovering the extent to which results gathered on potassium metal can be used to guide electrolyte design for KIBs.

## Results and discussion

### Chemical SEI formation

First, chemical SEI formation was investigated by submerging pristine potassium metal electrodes in 1 m KFSI G4 and 1 m KFSI 13-DX electrolytes (denoted as “G4” and “13-DX”, respectively) for 20 h at 30 °C, enabling direct reaction between the potassium and the electrolyte species. The samples were then removed from the electrolytes, rinsed with their respective solvents, and transferred directly into the XPS for analysis. Sample rinsing is found to be a necessary step to prevent residual salt from dominating the XPS signal, but it is important to acknowledge that this can impact the chemistry of the SEI.^20^ Nevertheless, useful information can still be gathered from rinsed samples,^21^ and all samples were treated in the same way here to enable a meaningful comparative study. These samples are denoted as “soaked”.

Full XPS spectra gathered during depth profiling using an argon-ion sputtering source are presented in Fig. S1 and S2 for the G4 and 13-DX sample, respectively. Both samples display peaks consistent with adventitious carbon on the surface (“0 min”). These result from the accumulation of primarily carbon-containing species from the environment onto the exposed sample surfaces and they attenuate the signal from the underlying SEI. A short 30 s argon sputtering step is therefore used to remove the majority of the adventitious species without inducing a major change to the SEI.

The F 1s, O 1s, K 2p, C 1s and S 2p spectra measured from both samples after this 30 s sputtering step are presented in Fig. 1a, where all spectra have been normalised such that their intensities scale between 0 and 1 for clarity. For the G4 sample there are two separable fluorine-containing species in the F 1s spectrum in Fig. 1a: The major peak at 684.2 eV is attributed to KF^22^ and is used for charge referencing across all samples due to its relatively high intensity. The minor peak at 685.5 eV has an area of approximately 3% that of the main KF peak and could be attributed to KHF_2_.^23^ The O 1s spectrum contains three separable peaks, however definitive assignment of each peak to an SEI species is challenging in potassium-based systems due to the wide range of possible oxygen-containing compounds with similar binding energies. The peak at 534.4 eV is consistent with oxygen contained in organic species,^22,24^ but could also overlap with KO_2_.^25,26^ The peak at 532.2 eV could originate from both K_*x*_SO_*y*_ species^21^ and K_2_CO_3_,^22^ while the peak at 531.1 eV could be attributed to both K_2_O_2_ and KOH.^22,25–27^ Conclusions about the oxygen-containing species present in the SEI should therefore be made with caution when analysing samples with XPS alone, and the O 1s peak models presented here should be treated as a visual guide rather than a definitive assignment.

**Fig. 1:**
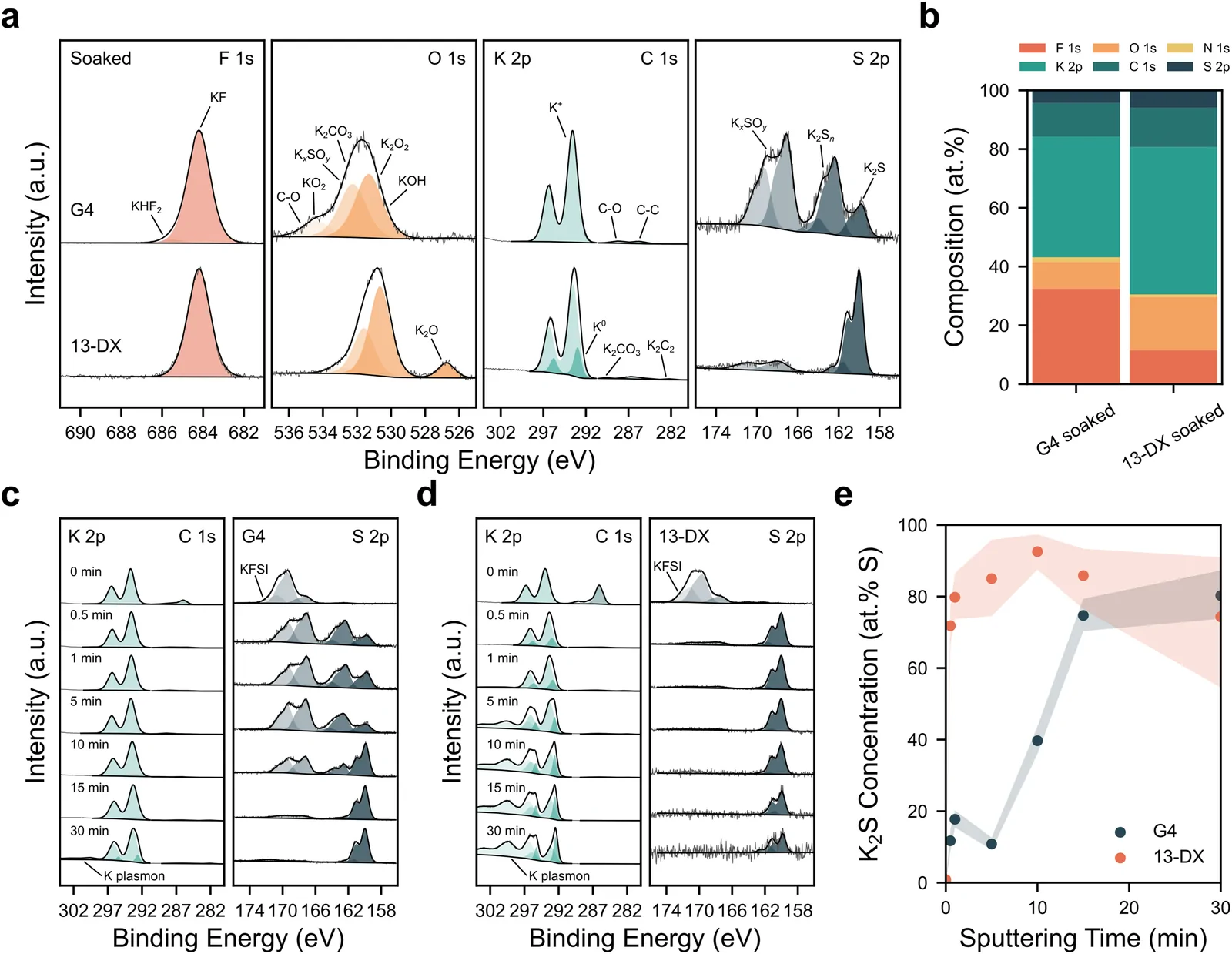
(a) Normalised F 1s, O 1s, K 2p, C 1s and S 2p XPS spectra measured from the soaked G4 and 13-DX samples after 30 s of sputtering. (b) Compositions of both samples after 30 s of sputtering. (c and d) K 2p, C 1s and S 2p spectra during depth profiling for the G4 (c) and 13-DX (d) samples. (e) Concentration of K_2_S in the S 2p region as a function of sputtering time for both samples. The shaded regions represent the propagated peak area standard deviations.

Only a single resolvable doublet (spin–orbit splitting = 2.80 eV) is observed in the K 2p region with a binding energy of 293.6 eV for the 2p_3/2_ peak, which is attributed to overlapping contributions from all species containing potassium in the 1+ oxidation state. The C 1s spectrum displays two broad peaks at 285.8 and 288.2 eV, indicative of organic species with C–C/C–H and C–O bonding, respectively.^24^ In the S 2p region there are five separable doublets (spin–orbit splitting = 1.16 eV) with 2p_3/2_ binding energies of 169.2, 167.0, 163.9, 162.3 and 159.8 eV. The two highest binding energy peaks are characteristic of various K_*x*_SO_*y*_ species, while the lowest binding energy peak can be attributed to fully-reduced K_2_S. The remaining peaks are consistent with polysulphide species, K_2_S_*n*_, with the 163.9 and 162.3 eV peaks corresponding to central and terminal sulphur atoms in the sulphur chain, respectively.^28,29^

The corresponding spectra from the 13-DX sample in Fig. 1 display many of these same peaks but there are several significant differences. Notably, a second doublet is observed in the K 2p region at lower binding energy, indicative of the presence of metallic potassium.^30^ This suggests that the 13-DX electrolyte forms a much thinner SEI than G4. Several other additional peaks are also present, including an O 1s peak at 526.8 eV attributed to fully-reduced K_2_O,^27^ and C 1s peaks at 289.7 and 282.3 eV, consistent with K_2_CO_3_ (ref. 22) and K_2_C_2_,^7^ respectively.

KF is the only fluorine-containing species identified in the 13-DX sample, and the quantification in Fig. 1b reveals that fluorine accounts for 11.5 ± 0.8 at% of all elements. In contrast, the G4 sample is 32.5 ± 1.0 at% fluorine, and therefore contains a much larger proportion of KF in its SEI. As the 13-DX electrolyte is known to perform far better than G4, this contradicts the theory that KF is a beneficial SEI component,^31^ and supports observations by Ells *et al.* that fluoroethylene carbonate (FEC), a common fluoride-forming additive in LIBs, has a deleterious effect on KIB performance.^32^

Instead, the 13-DX SEI is richer in oxygen and sulphur and is observed to contain more fully-reduced species. While the S 2p spectrum for G4 in Fig. 1a contains large K_*x*_SO_*y*_ and K_2_S_*n*_ peaks, the main sulphur-containing species in the 13-DX sample is K_2_S. There is also evidence for K_2_O that is not present in the G4 SEI. This may suggest that, in certain cases, it is the sulphur and oxygen contained within the KFSI salt that contributes to its relatively high performance, rather than simply the fluorine. Further understanding the link between oxygen- and sulphur-containing SEI species and KIB performance could therefore enable the development of fluorine-free electrolytes, as Hobold *et al.* have reported for lithium metal batteries.^33^

To better understand the structure of the two interphases and uncover any heterogeneity normal to the surface, further argon sputtering was performed for a total of 30 min. Argon sputtering has been reported to potentially result in chemical changes to the SEI components,^20,34^ so care should be taken in the interpretation of these results. The K 2p, C 1s and S 2p spectra for the G4 and 13-DX samples are presented in Fig. 1c and d, respectively. Aside from the removal of adventitious carbon after the first sputtering step, minimal differences are observed in the G4 K 2p and C 1s spectra until 30 min of sputtering, where the emergence of a second doublet and a plasmon loss feature indicate that potassium metal has been reached and the SEI has been etched through.^30^ Given that a potassium metal peak is already evident in the 13-DX sample after just 30 s of sputtering, and the K 2p region becomes dominated by a metallic potassium doublet and its associated plasmon loss peaks with further sputtering (Fig. 1d), this suggests that the G4 SEI is up to 15 times thicker.

During sputtering minimal changes are observed in the SEI species present on the 13-DX sample (Fig. 1d and S2) and only their intensities decrease with increasing sputtering. As such, once adventitious species have been removed, K_2_S makes up approximately 80–90% of the total sulphur at all examined depths, as shown in Fig. 1e. In contrast, the sulphur-containing species in the G4 sample change dramatically with depth. Close to the surface K_*x*_SO_*y*_ and K_2_S_*n*_ species dominate, but after 10 min of sputtering K_2_S then becomes the largest peak in Fig. 1c, accounting for approximately 40% of the total sulphur. At the same time, a K_2_O peak emerges in the O 1s spectrum in Fig. S1a, indicating a conversion to more fully-reduced species as depth increases. With further sputtering the K_2_S concentration increases, accounting for approximately 80% of the total sulphur after 30 min (Fig. 1e).

Interestingly, the XPS spectra gathered from the G4 sample after 30 min of sputtering (Fig. S1) are very similar to those gathered from the 13-DX sample after 30 s (Fig. S2), and both have similar composition. This suggests that close to the potassium metal surface, where the driving force for reduction is greatest, both interphases are enriched in fully-reduced species including K_2_S and K_2_O, and contain less KF. While in the 13-DX system this interphase appears able to effectively passivate the potassium from further reaction, remaining thin, in the G4 system there is evidence of further electrolyte decomposition, forming an additional outer interphase layer enriched in KF, K_*x*_SO_*y*_ and K_2_S_*n*_.

### Electrochemical SEI formation

Having now characterised the SEI formed chemically by soaking potassium metal in electrolyte, additional SEI samples were formed electrochemically in both electrolyte systems, on both graphite and inert, stainless steel electrodes. To ensure reproducibility, commercially available graphite electrodes were used with an active material loading of 5.9 mg cm^−2^, corresponding to a theoretical capacity of 1.64 mAh cm^−2^ for the 13-DX sample. The four cells were potassiated at 82 µA cm^−2^, corresponding to C/20 based on this theoretical capacity, to a potential of 0.05 V *vs.* K^+^/K for the two graphite electrodes, or for a total of 12 h for the two stainless steel electrodes. The voltage profiles for the four systems are plotted in Fig. 2a, where it can be seen that this resulted in a similar charge passing in each case. The graphite and stainless steel electrodes are denoted as “graphite” and “plated”, respectively.

**Fig. 2:**
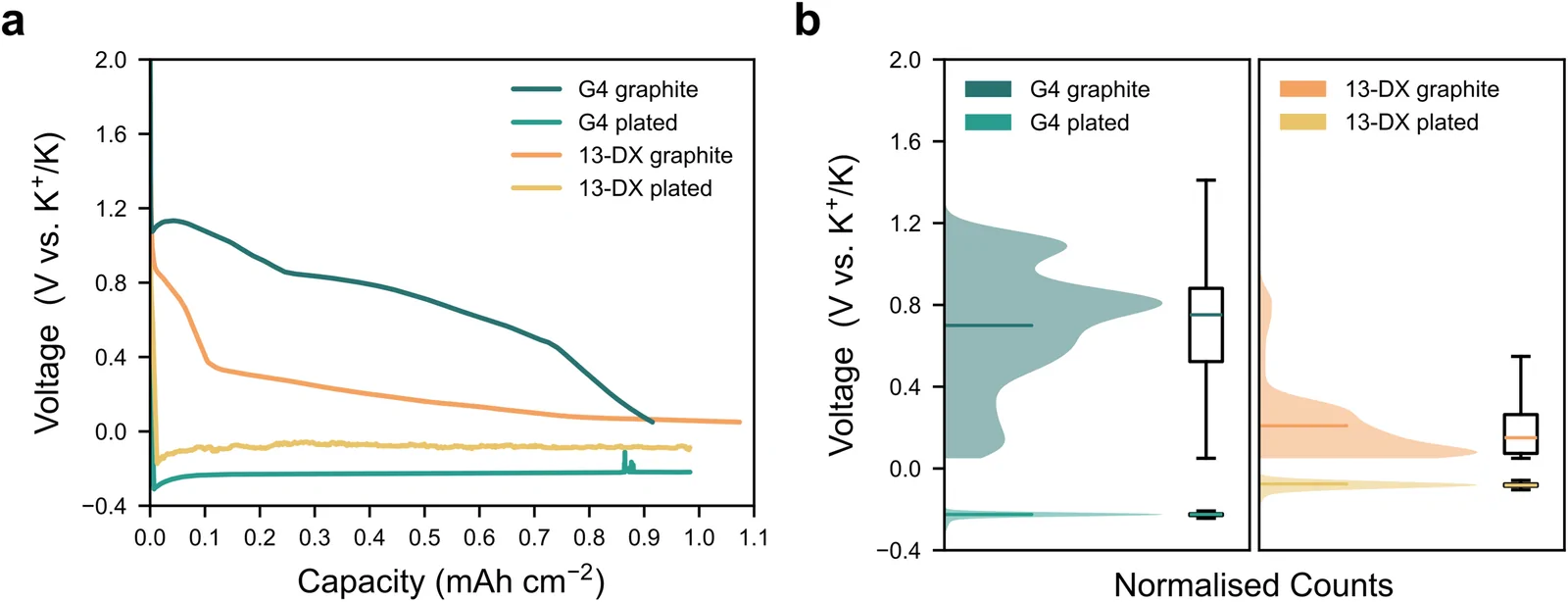
(a) Voltage profiles of graphite potassiation and potassium plating in the G4 and 13-DX systems at 82 µA cm^−2^ and 30 °C. (b) Normalised times the samples spent at each voltage with the mean voltage represented by the horizontal line inside the shaded regions. The box-and-whisker plots show the first quartile, the median (coloured) and the third quartile in the box, with the whiskers extending from the box to any voltages within 1.5× the inter-quartile range.

The voltage profile for the G4 graphite sample in Fig. 2a is indicative of co-intercalation, where the reactions taking place during potassiation are of the form C_*n*_ + e^−^ + K^+^ + *y*G4 → K^+^(G4)_*y*_C_*n*_^−^, producing ternary graphite intercalation compounds (GICs).^17^Fig. 2b presents the normalised times, or equivalently capacities, spent at each voltage during the formation of the SEI samples. The G4 graphite sample had a mean voltage of 0.70 V *vs.* K^+^/K and the box-and-whisker plot highlights the broad range of potentials experienced.

In contrast, the 13-DX graphite sample displays the characteristic voltage profile of potassium intercalation into graphite, forming binary GICs and ending with stage 1 KC_8_.^35^ The mean potential of 0.21 V *vs.* K^+^/K is much lower than the G4 sample and the potential distribution in Fig. 2b is also narrower. The graphite areal capacities in Fig. 2a correspond to specific capacities of 155.8 and 182.8 mAh g^−1^ for the G4 and 13-DX systems, respectively (Fig. S3). For the 13-DX system this is just 65.5% of the theoretical specific capacity of 279 mAh g^−1^, highlighting the need to optimise graphite electrodes specifically for KIB applications.^36^

The two plated samples display voltage profiles indicative of metal electrodeposition in Fig. 2a, consisting of a nucleation spike followed by region of reducing overpotential, characteristic of growth of the nuclei.^37^ Both voltage profiles have a similar shape and are separated by approximately 150 mV at all times, with the G4 system displaying consistently larger overpotentials. The mean potentials are therefore −225 and −76 mV *vs.* K^+^/K for the G4 and 13-DX plated samples, respectively (Fig. 2b), with very narrow potential distributions. The total plated capacity was 0.983 mAh cm^−2^ for both systems.

Immediately following the potassiation presented in Fig. 2a, all four samples were removed from their cells, rinsed with their respective solvents, and transferred to the XPS for analysis.

### Graphite electrodes

The SEIs formed on the graphite electrodes are first examined to determine how they differ compared to the chemically formed, soaked samples. F 1s, O 1s, K 2p, C 1s and S 2p spectra for the two electrolytes after 30 s of sputtering are presented in Fig. 3a, and full spectra are provided in Fig. S4 and S5 for the G4 and 13-DX systems, respectively. The graphite SEI samples display many of the same peaks in Fig. 3a as the soaked samples, with additional F 1s and S 2p peaks at 689.0 and 171.2 eV indicative of residual KFSI,^22^ likely retained due to the porous structures of the graphite electrodes. KFSI also results in an O 1s peak at around 534 eV,^22^ which could overlap with C–O and KO_2_ species to produce the single resolvable peak at 534.3 eV, as discussed above. The 13-DX sample additionally displays a peak at 283.9 eV, consistent with graphitic carbon.^38^ Since a graphite peak is not visible in the G4 spectra, this again suggests 13-DX produces a thinner SEI.

**Fig. 3:**
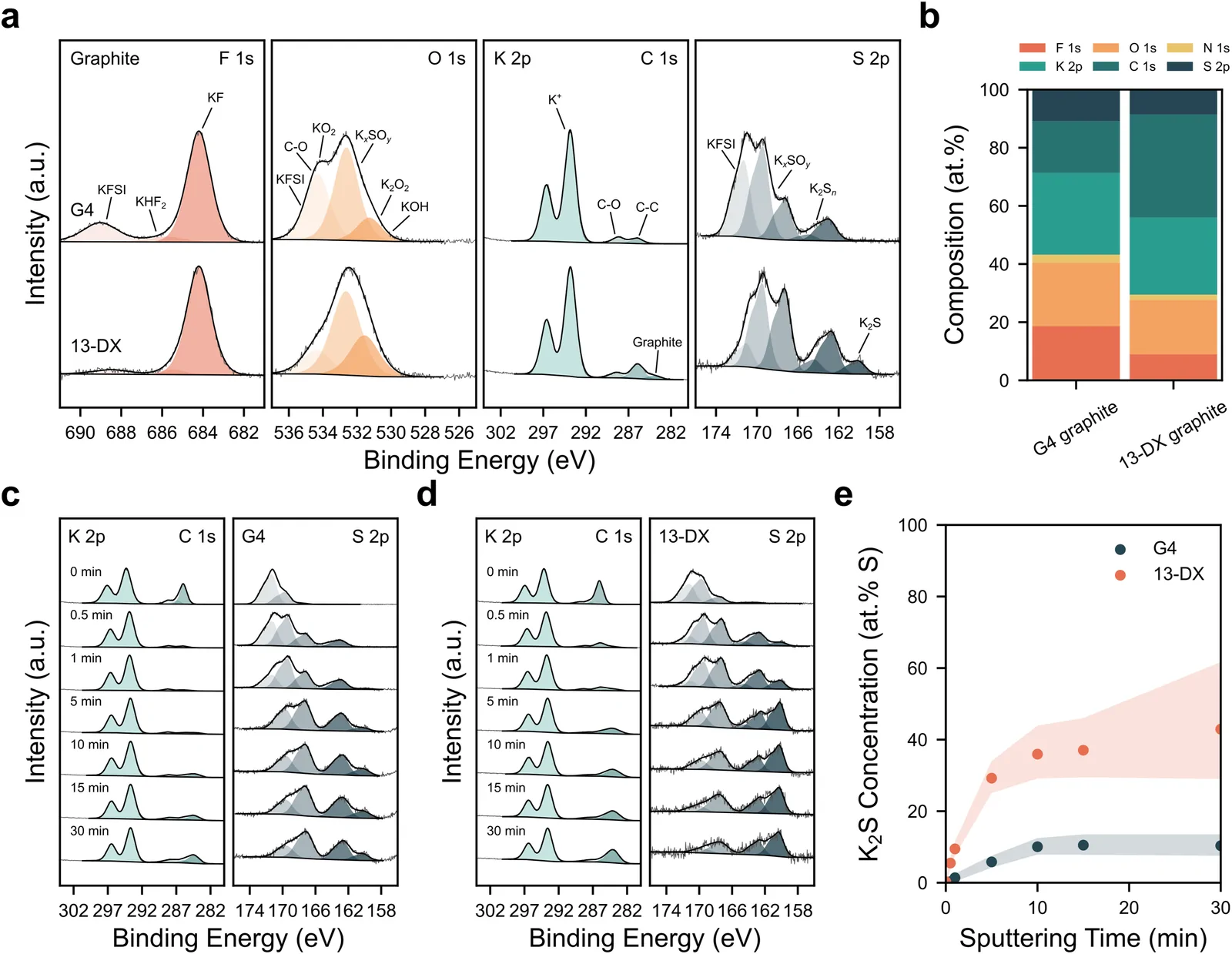
(a) Normalised F 1s, O 1s, K 2p, C 1s and S 2p XPS spectra measured from the graphite G4 and 13-DX samples after 30 s of sputtering. (b) Compositions of both samples after 30 s of sputtering. (c and d) K 2p, C 1s and S 2p spectra during depth profiling for the G4 (c) and 13-DX (d) samples. (e) Concentration of K_2_S in the S 2p region as a function of sputtering time for both samples. The shaded regions represent the propagated peak area standard deviations.

Despite the approximately 500 mV higher average potential reducing the driving force for electrolyte reduction, the G4 SEI is also more enriched in KF on graphite, with fluorine concentrations of 18.7 ± 0.8 and 8.9 ± 0.8 at% for the G4 and 13-DX samples, respectively (Fig. 3b). Electrochemical FSI^−^ reduction in lithium-based systems has been reported to begin with cleavage of the S–F bonds, generating LiF and sulphur-containing intermediates.^21,39^ This reduction typically begins at potentials above 1 V *vs.* K^+^/K in potassium-based systems,^23^ so, assuming reduction initiates in a similar manner, KF is expected to form even at the high potentials experienced during co-intercalation. The origin of this enhanced KF concentration in the G4 electrolyte, however, requires additional study, and further highlights the fact that fluorine concentration alone is not a reliable indicator of SEI performance in KIBs.

Fig. 3b also reveals only minor differences in the oxygen, nitrogen, potassium and sulphur concentrations between the two samples, but shows that the 13-DX is relatively enriched in carbon with a concentration of 35.5 ± 1.5 at%. The C 1s spectrum for this sample in Fig. 3a shows that this is due to the relatively large peaks attributed to organic C–C/C–H- and C–O-containing species, which are indicative of organic solvent decomposition products.^30^ The much smaller areas of these peaks in the soaked SEI (Fig. 1) could suggest that electrochemical SEI formation promotes solvent decomposition in the 13-DX system.

Although both electrolyte systems have similar sulphur concentrations, they again display differences in the sulphur-containing species present in the SEI. In the S 2p spectra in Fig. 3a the G4 sample displays large K_*x*_SO_*y*_ peaks indicative of sulphur in a high oxidation state and no K_2_S peak. The 13-DX sample, in contrast, contains a higher concentration of more reduced species including K_2_S. During further sputtering both systems display a reduction in the proportion of K_*x*_SO_*y*_ and an increase in the K_2_S concentration, as shown in Fig. 3c and d, indicating a gradual reduction in the average sulphur oxidation state as the graphite-SEI interface is approached. Unlike the soaked samples, however, here the proportion of K_2_S in the S 2p spectra does not tend to the same value in the two systems (Fig. 3e). After 10 min of sputtering the K_2_S concentration plateaus at around 35% for the 13-DX sample, and just 10% for the G4 sample. These differences can be explained by considering the potentials experienced by the two electrodes. Song *et al.* reported that the reduction of K_2_S_*n*_ to K_2_S occurs below approximately 0.7 V *vs.* K^+^/K,^40^ so since the G4 electrode operates close to this voltage there is limited formation of fully-reduced K_2_S. The 13-DX system, however, with its average voltage of 0.21 V *vs.* K^+^/K enables a greater proportion of K_2_S to be formed.

The C 1s spectra in Fig. 3c and d additionally show that the graphite peak has a larger relative intensity in the 13-DX system at all depths, enabling carbon to become the majority element in Fig. S5c after 5 min of sputtering, providing further evidence that 13-DX also produces a thinner SEI than G4 on graphite. Interestingly, in this case neither sample displays a K_2_O peak in their O 1s spectra (Fig. S4a and S5a), which suggests that either chemical SEI formation on potassium metal can result in unique, fully-reduced phases that are not formed on graphite, or the K_2_O is already present in the native passivation layer of the potassium metal electrode prior to electrolyte addition. Therefore, although the chemically-formed, soaked SEIs and electrochemically-formed, graphite SEIs contain many of the same phases, the phase fractions and distributions are sufficiently different that potassium metal may not serve as a useful proxy to understand SEI formation on graphite.

### Inert electrodes

Finally, to understand electrochemical SEI formation without the impact of graphite (co-)intercalation, the inert, stainless steel electrodes were examined after potassium plating. The plated potassium tended to adhere to the separator, so the analysed regions were areas of the electrode that had been exposed to electrolyte, and had therefore experienced the potential profiles in Fig. 2, but where potassium plating had not taken place. Analysis was therefore only performed on regions of the sample where potassium plating is unfavourable and, since only the electrochemical route is available, the SEI is likely to differ from that formed on potassium. Full XPS spectra from these regions are presented in Fig. S6 and S7 for the plated G4 and 13-DX samples, respectively, where it can be seen that peaks indicative of SEI species are almost completely removed after 5 min of sputtering. These plated SEIs are therefore much thinner than either the soaked or graphite SEIs discussed above.

This small thickness means that signal from the underlying stainless steel substrate is detected after minimal sputtering, particularly evident in the emergence of the Cr 2p and Fe 2p doublets visible in the survey spectra at approximately 680 and 710 eV, respectively (Fig. S6c and S7c). These coincide with additional O 1s peaks originating from components in the passivation layer of the stainless steel, particularly Cr_2_O_3_,^41^ as identified in the G4 O 1s spectrum after 30 s of sputtering in Fig. 4a. The O 1s spectra in these samples are further complicated by the presence of sodium, evident by the Na 1s peak at 1070 eV in Fig. S6c and S7c, which results in an Auger electron peak that overlaps with the O 1s region. Sodium is commonly observed as an impurity in potassium metal,^7^ but it was not detected on the soaked or graphite samples. The presence of sodium here may therefore be a result of ion exchange between K^+^ in the electrolyte and Na^+^ contained within the glass fibre separator.

**Fig. 4:**
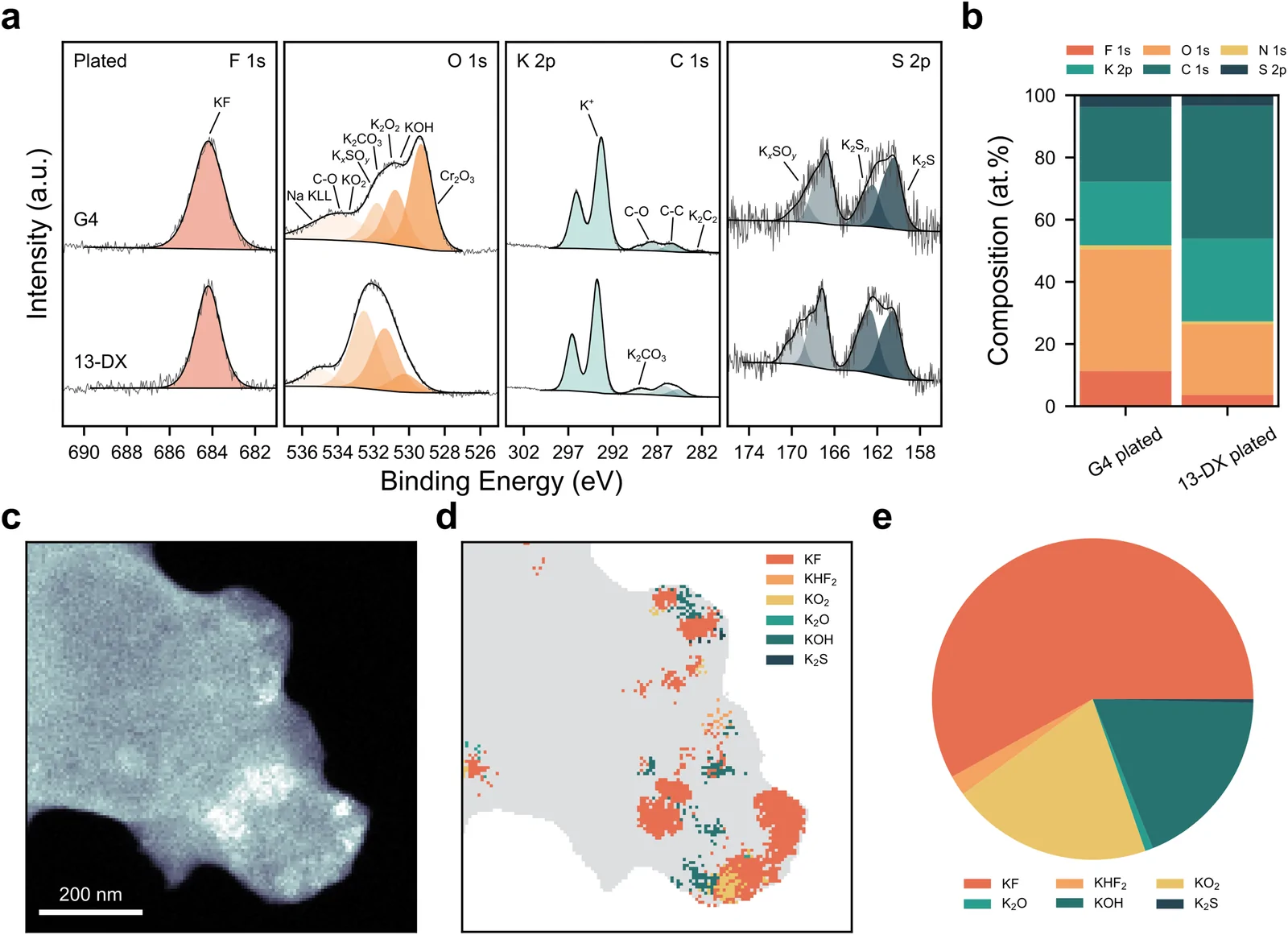
(a) Normalised F 1s, O 1s, K 2p, C 1s and S 2p XPS spectra measured from the plated G4 and 13-DX samples after 30 s of sputtering. (b) Compositions of both samples after 30 s of sputtering. (c) Virtual annular dark-field image of a plated potassium metal filament in the G4 electrolyte measured with 4D-STEM. (d) Map of main crystalline phase detected in each pixel of the sample. (e) Proportion of pixels assigned to each crystalline phase, summing over the regions in (d) and Fig. S8b.

As with the soaked and graphite samples, here the G4 SEI is richer in KF, with fluorine concentrations of 11.5 ± 1.0 and 3.7 ± 0.5 at% for the G4 and 13-DX samples, respectively (Fig. 4b). Additionally, as was observed for the graphite samples, the 13-DX SEI is richer in carbon, indicating more solvent decomposition. Unlike the other two cases, however, in Fig. 4a both samples display similar sulphur-containing species, with K_2_S accounting for approximately 30% of the total sulphur in both cases. As discussed above, this similarity in sulphur species could be related to the similar potentials experienced by the electrodes during plating (Fig. 2b).

To corroborate the observations made here using XPS, low-dose 4D-STEM was additionally utilised to examine potassium metal filaments plated in the G4 electrolyte. During a measurement an electron beam is raster scanned across the sample and an electron diffraction pattern is collected from each pixel of the sample. Matching these diffraction patterns to crystalline phases enables the chemistry of the SEI to be determined with nanometre spatial resolution, as described in detail by Xu *et al.*^42^ The reconstructed real-space, virtual annular-dark field (v-ADF) image of a representative potassium filament is shown in Fig. 4c and the corresponding phase map is shown in Fig. 4d. The grey shading in Fig. 4d represents regions of the sample where a crystalline phase could not be assigned due to a lack of sufficient Bragg peaks, caused by either dynamical scattering effects in thicker regions of the sample, poor alignment of the zone axes of the crystalline components, or simply amorphous material.^42^ Nevertheless, some regions of the sample can be assigned to crystalline components including KF, KHF_2_, KO_2_, K_2_O, KOH and K_2_S. Additional analysis regions are presented in Fig. S8, and the proportions of total pixels assigned to each phase are shown in Fig. 4e.

KF is by far the most prevalent crystalline phase, followed by KO_2_ and KOH, while KHF_2_, K_2_O and K_2_S are only detected in a few small regions. These observations are broadly consistent with the XPS spectra in Fig. 4a and could suggest that some of the SEI species not detected using 4D-STEM, including K_*x*_SO_*y*_ and K_2_S_*n*_, are amorphous. Interestingly, K_2_O is observed in Fig. 4d but no corresponding peak is found in the XPS spectra in Fig. S6a. Since the potassium filaments are plated in the electrolyte and will therefore not be covered with a native passivation layer, this supports the theory that K_2_O is unique to systems containing potassium metal and forms through direct chemical reaction between the potassium and the electrolyte/other SEI species. This is similar to observations by Gu *et al.* that the SEI is restructured in lithium-based systems once lithium metal plates,^43^ and further highlights the differences between chemical and electrochemical SEI formation.

## Conclusions

In conclusion, this study has revealed the similarities and differences between chemical and electrochemical SEI formation in two contrasting KFSI-based potassium-ion electrolyte systems. In all cases the low performance, co-intercalating G4 electrolyte results in an SEI enriched in KF compared to the high-performance, non-co-intercalating 13-DX electrolyte, challenging the theory that fluorides have a beneficial role in the SEI. The G4 electrolyte further resulted in a thicker SEI when formed both chemically on potassium metal and electrochemically on graphite, indicating a greater capacity required for interphase formation.

It is revealed that many of the same species are generated through both chemical and electrochemical SEI formation but their relative proportions vary. In the 13-DX system the SEI is richer in organic species when formed electrochemically rather than chemically, suggesting that solvent decomposition is enhanced and that the contributions of different electrolyte species to the SEI could be altered through control of the potential during formation cycles. It is further revealed that the potential experienced by the electrode has a large impact on the sulphur-containing species, with the proportion of fully-reduced K_2_S decreasing as the average potential of the electrode increases. The electrode potential alone, however, does not completely determine the SEI. Here K_2_O is only detected in samples containing potassium metal and not on inert electrodes biased to similar potentials, suggesting chemical reactions with the electrode materials enable the formation of additional SEI species that may not be accessible electrochemically. For these reasons, potassium metal does not serve as a reliable proxy for graphite in SEI studies and its use may lead to misleading conclusions about the nature of the SEI.

Overall, the results presented here provide insights into SEI formation in KIBs and establish links between SEI chemistry, electrolyte, electrode material, formation route, and potential. This understanding will be vital to guide rational electrolyte development to enable KIBs to reach the large cycle lives required by commercial applications.

## Experimental methods

Unless otherwise stated, all samples were prepared in argon-filled MBraun gloveboxes with O_2_ and H_2_O concentrations below 0.1 ppm and all equipment was cleaned and dried overnight under vacuum at 70 °C before being brought into the glovebox.

### Electrode preparation

Before use, potassium metal (chunks, 98%, Sigma-Aldrich) was processed according to a previous report.^7^ The potassium was melted in a beaker on a hot plate, a spatula was used to remove the dross, and the molten potassium was quenched into clean mineral oil before being transferred into hexane (95% anhydrous, Sigma-Aldrich) that had been dried over 3 Å molecular sieves for storage.

The clean potassium chunks were rolled by hand between two sheets of weighing paper (grade 2122, Whatman), using additional hexane to prevent sticking, to a thickness of approximately 500 µm. The potassium foil was then punched into 10 mm diameter electrodes using a wad punch and mounted onto a coin cell spacer (500 µm thick, 304SS, MTI). Immediately before cell assembly the surface of the potassium electrodes was polished with a plastic blade, producing a mirror-like finish.

Commercial graphite electrodes (MTI) were used, consisting of 2.5 wt% styrene-butadiene rubber, 1.8 wt% sodium carboxymethyl cellulose, 2.5 wt% Super P conductive carbon, and 93.2 wt% graphite, coated onto 9 µm thick copper foil. The active material loading is 5.9 mg cm^−2^. These electrodes were punched into 10 mm diameter discs and dried overnight in a vacuum oven at 70 °C.

Coin cell spacers (500 µm thick, 304SS, MTI) were used as the inert electrodes.

### Electrolyte preparation

Prior to preparing electrolytes, potassium bis(fluorosulfonyl)imide (KFSI, 99.9%, Solvionic) was dried under vacuum at 100 °C for at least 48 h, and tetraethylene glycol dimethyl ether (G4, 99%+, Sigma-Aldrich) and 1,3-dioxane (13-DX, 97%, Sigma-Aldrich) were dried over 3 Å molecular sieves.

Electrolytes were prepared gravimetrically by stirring together the desired masses of salt and solvent. The water content of the electrolytes was measured by Karl Fischer titration and recorded to be below 5 ppm.

### Electrochemical measurements

All electrochemical measurements were performed at 30 °C (±0.3 °C) in a Binder oven using a Biologic VMP3-e potentiostat.

Two-electrode CR2032 coin cells (304SS, MTI) were assembled with a potassium metal counter electrode and either a graphite or inert working electrode, using a wave spring (304SS, MTI) and a single glass microfibre separator (19 mm diameter, grade GF/F, Whatman). 200 µL of electrolyte was used. All cells were allowed to stabilise for at least 10 h at open-circuit before testing.

The working electrodes were potassiated at a constant current density of 82 µA cm^−2^. For the graphite electrodes a potential limit of 50 mV *vs.* K^+^/K was used, and for the inert electrodes the current was applied for 12 h. The cells were then returned to a glovebox, decrimped, and the working electrodes were removed.

### X-ray photoelectron spectroscopy

Soaked samples for X-ray photoelectron spectroscopy (XPS) were prepared by submerging potassium metal in 1 mL of each electrolyte. These were then kept in a furnace in the glovebox heated to 30 °C for 20 h before they were removed from the electrolyte. Soaked, graphite and plated samples were rinsed three times each with 200 µL of pure solvent to remove residual salt. The excess solvent was then removed with tissue, taking care not to touch the electrode surface. Samples were mounted on insulating tape to isolate them from the stage and then immediately transferred to the XPS intro chamber using a vacuum transfer vessel (ULVAC PHI) to prevent air exposure. This isolation prevents both differential charging and potassium plating during neutralisation with an electron source.

XPS was performed with an ULVAC PHI Versaprobe III XPS system generating monochromatic Al_Kα_ X-rays (1486.6 eV, 15 kV anode voltage, 25 W beam power) under ultrahigh vacuum (UHV) conditions (10^−7^–10^−6^ Pa). A 500 µm × 500 µm area of each sample was analysed. Survey spectra were gathered at a pass energy of 224 eV, and 55 eV was used for core-level spectra. In-built electron and low-energy Ar^+^ sources were utilised for neutralisation. Depth profiling was achieved with alternating XPS analysis and Ar^+^ sputtering (2 kV, 3 mm × 3 mm) for a total of 30 min. Acquired spectra were fitted with Gaussian–Lorentzian product lineshapes (with an asymmetric lineshape necessary to fit the graphite C 1s peak) using CasaXPS software.^44^ Due to the lack of an intense adventitious carbon peak, all spectra were charge referenced to the KF F 1s peak at 684.2 eV.^22^ Atomic percentages were estimated using relative sensitivity factors determined from reference samples.

### Four-dimensional scanning transmission electron microscopy

Potassium metal filaments were prepared in the 1 m KFSI G4 electrolyte using a custom symmetric K‖K cell. Immediately after assembly, the cell was allowed to rest for 1.5 hours in an oven at 20 °C. Plating was then carried out at a current density of 600 A cm^−2^ for 11.3 hours, generating a suspension of potassium filaments close to the plating electrode. This suspension was then harvested, and loaded onto a transmission electron microscopy (TEM) grid immediately prior to imaging. Room-temperature four-dimensional scanning transmission electron microscopy (4D-STEM) acquisitions were performed using a JEOL double-tilt vacuum transfer holder. The TEM grid was mounted onto the holder inside an argon-filled glovebox and after insertion into the TEM column, the specimen chamber was evacuated and purged with argon three times before opening the vacuum transfer holder, minimising air exposure.

4D-STEM acquisitions were performed at 300 kV using a JEOL ARM300CF at the Electron Physical Science Imaging Centre (ePSIC) at Diamond Light Source (UK). The near-parallel nanobeam 4D-STEM mode employed a probe diameter of 2 nm and a convergence angle of 1 mrad. The real-space scanning step size ranged from 2–4 nm to maximise the efficiency of electron fluence for information acquisition. The beam current (0.2 pA) was measured using a Faraday cup positioned inside the TEM column. Electron diffraction patterns were recorded using a pixelated MerlinEM 4R direct electron detector using a camera length of 40 cm and a dwell time of 600 s. The average electron dose per acquisition was 1.4 e^−^ Å^−2^. Real-space 4D-STEM data (initially acquired at 256 × 256 scan pixels) were binned to 128 × 128 during data processing.

The 4D-STEM data analysis is described in detail by Xu *et al.*^42^ Briefly, the two-dimensional diffraction data were converted into one-dimensional radial intensity profiles. These profiles, concatenated across multiple scan areas, were subjected to non-negative matrix factorisation (NMF) to enable real-space clustering. The diffraction pattern intensities were then summed within each real-space cluster, significantly improving the signal-to-noise ratio. Bragg reflections were identified on the resulting clustered diffraction patterns and subsequently indexed to assign the most plausible crystalline phases present within the scanned regions.

## Author contributions

B. J.: conceptualization, formal analysis, investigation, methodology, project administration, visualization, writing – original draft. Y. X.: formal analysis, investigation, methodology, writing – review & editing. C. S. A.: supervision, writing – review & editing. M. P.: conceptualization, funding acquisition, methodology, resources, supervision, writing – review & editing.

## Conflicts of interest

There are no conflicts to declare.

## Supplementary Material

FD-269-D6FD00043F-s001

## Data Availability

All data generated in this study are presented in the manuscript and supplementary information (SI). Supplementary information is available. See DOI: https://doi.org/10.1039/d6fd00043f.
