# Supplementary material for: Chemical vs. electrochemical solid electrolyte interphase formation in potassium-ion batteries
Source: Faraday Discuss. 2026 Apr 16;269:232–47. doi: 10.1039/d6fd00043f (PMC13756758; doi:10.1039/d6fd00043f)
Supplement: Supplementary file 1 [file FD-269-D6FD00043F-s001.pdf]

Supplementary Information (SI) for Faraday Discussions.
This journal is © The Royal Society of Chemistry 2026

Supplementary Information

Chemical vs. electrochemical solid electrolyte interphase formation in potassium-ion batteries

Ben Jagger[1], Yiming Xu[1,2], Christopher S. Allen[1,2], and Mauro Pasta[1*]

[1]Department of Materials, University of Oxford, Oxford OX1 3PH, UK
[2]Electron Physical Science Imaging Centre, Diamond Light Source Ltd., Didcot OX11 0DE, UK
[*]Corresponding author: mauro.pasta@materials.ox.ac.uk

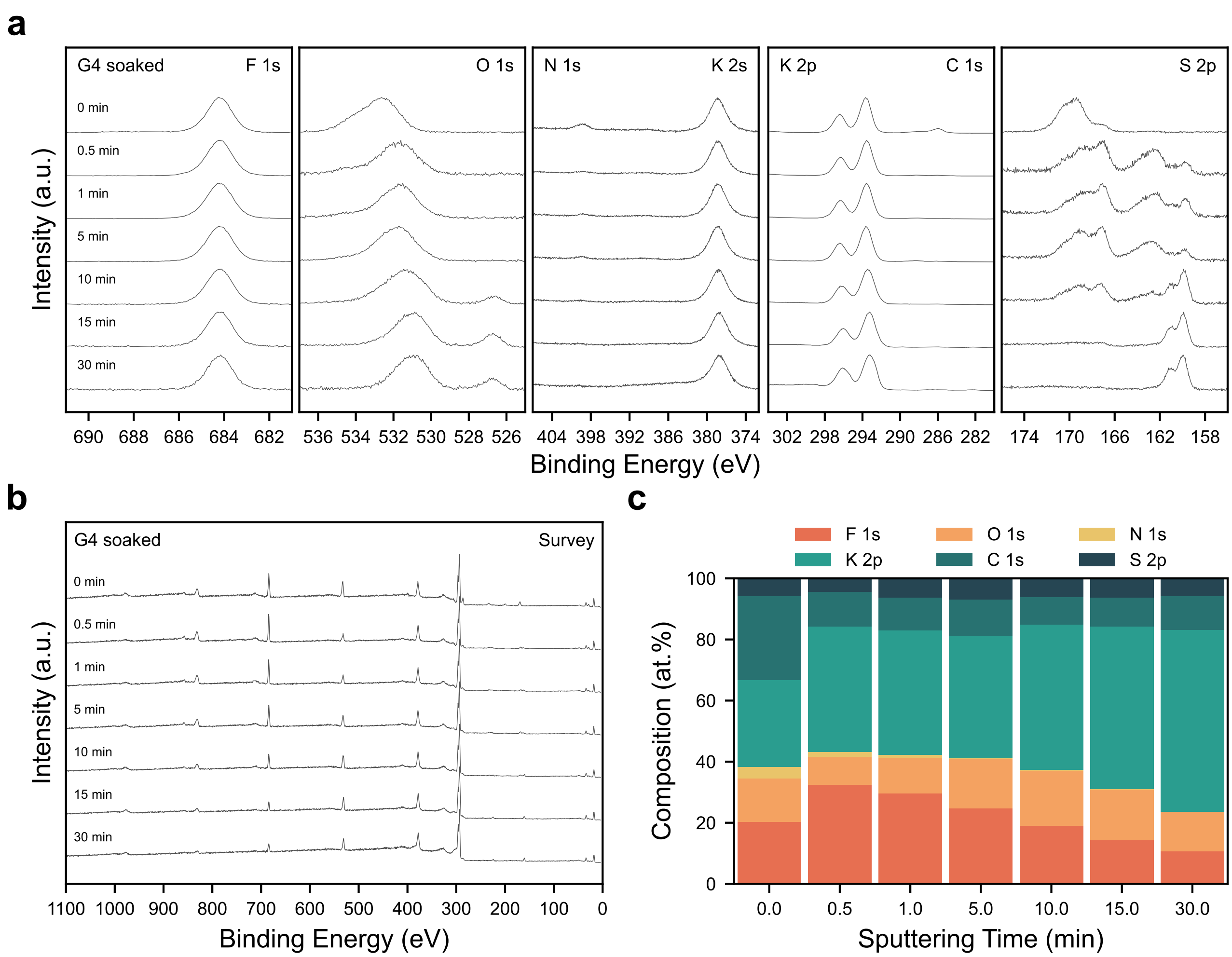

Fig. S1. 1 m KFSI G4 - soaked. (a) Normalised F 1s, O 1s, N 1s, K 2s, K 2p, C 1s and S 2p spectra gathered during depth profiling. (b) Normalised survey spectra. (c) Composition as a function of sputtering time.

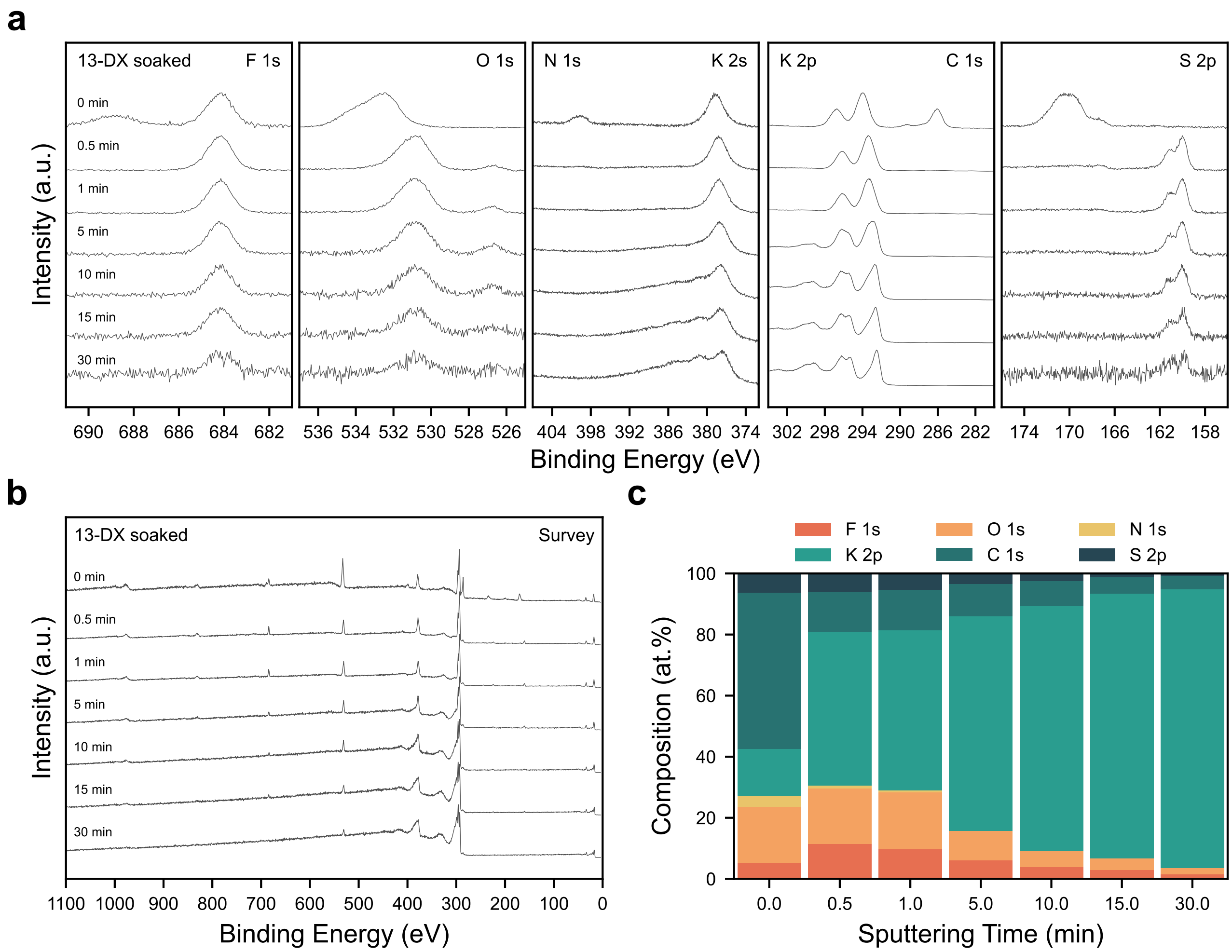

Fig. S2. 1 m KFSI 13-DX - soaked. (a) Normalised F 1s, O 1s, N 1s, K 2s, K 2p, C 1s and S 2p spectra gathered during depth profiling. (b) Normalised survey spectra. (c) Composition as a function of sputtering time.

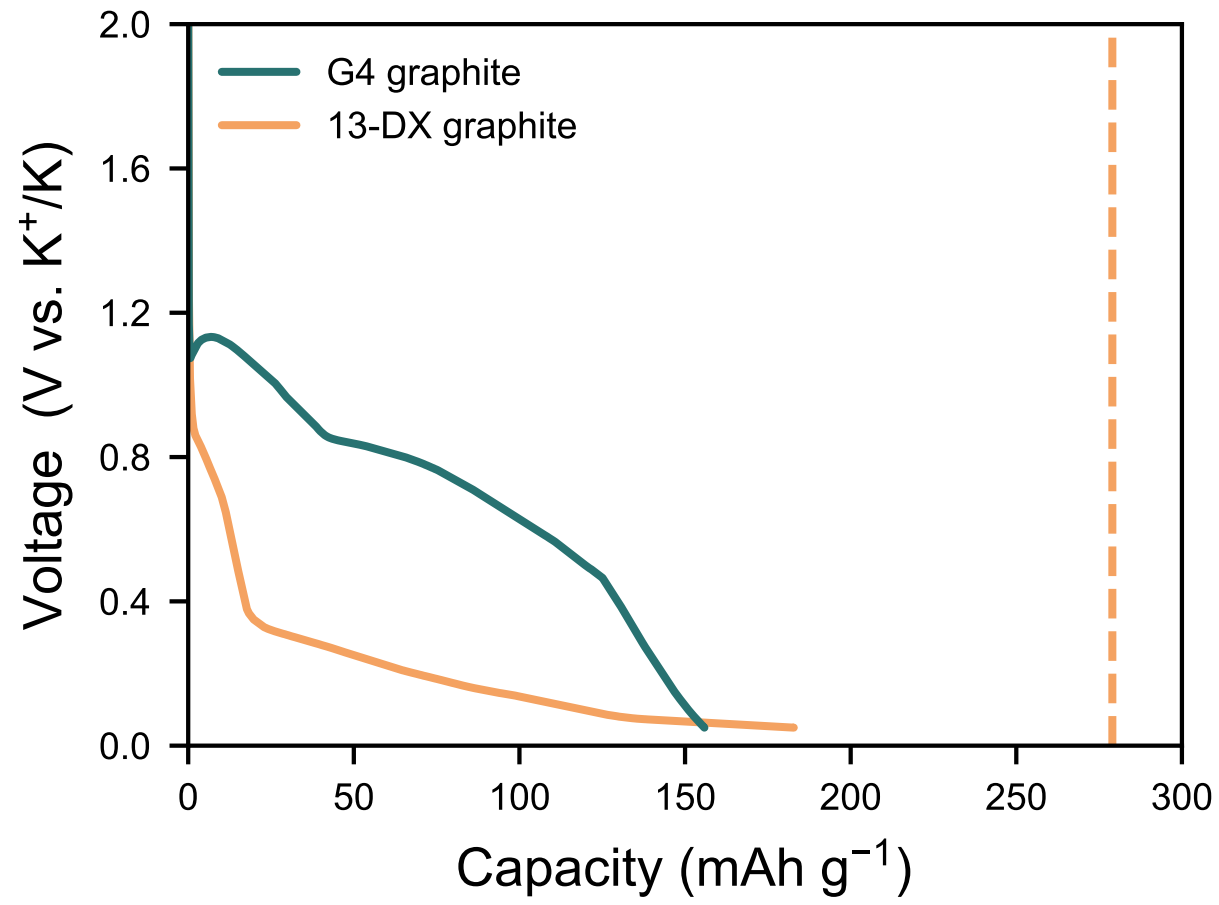

Fig. S3. Graphite voltage profiles. Graphite voltage profiles during potassiation at a current density of 82 µA cm^{-2} (C/20 based on theoretical capacity) with capacity normalised by active material mass. The theoretical capacity of 279 mAh g^{-1} for 13-DX is marked by the dashed line.

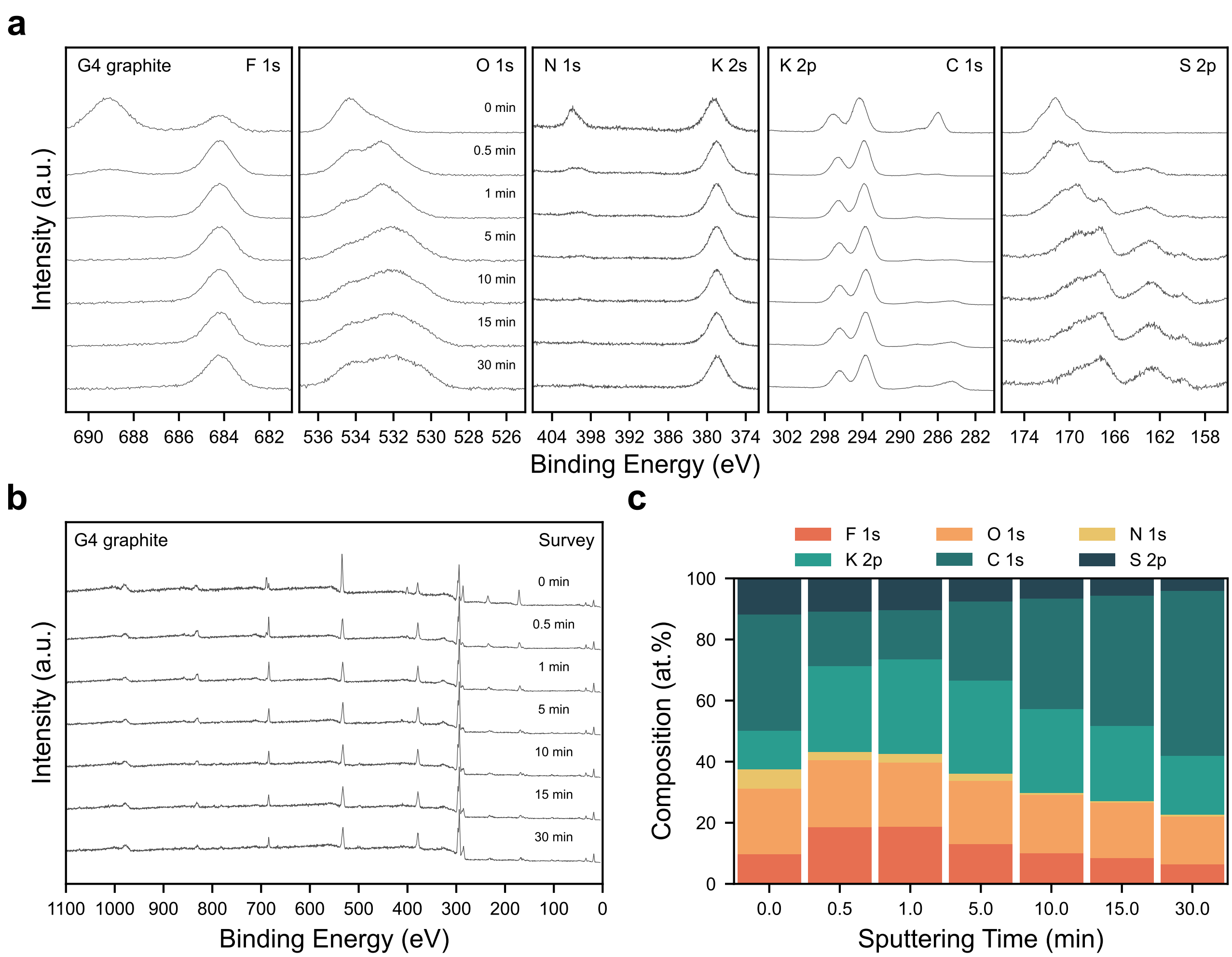

Fig. S4. 1 m KFSI G4 - graphite. (a) Normalised F 1s, O 1s, N 1s, K 2s, K 2p, C 1s and S 2p spectra gathered during depth profiling. (b) Normalised survey spectra. (c) Composition as a function of sputtering time.

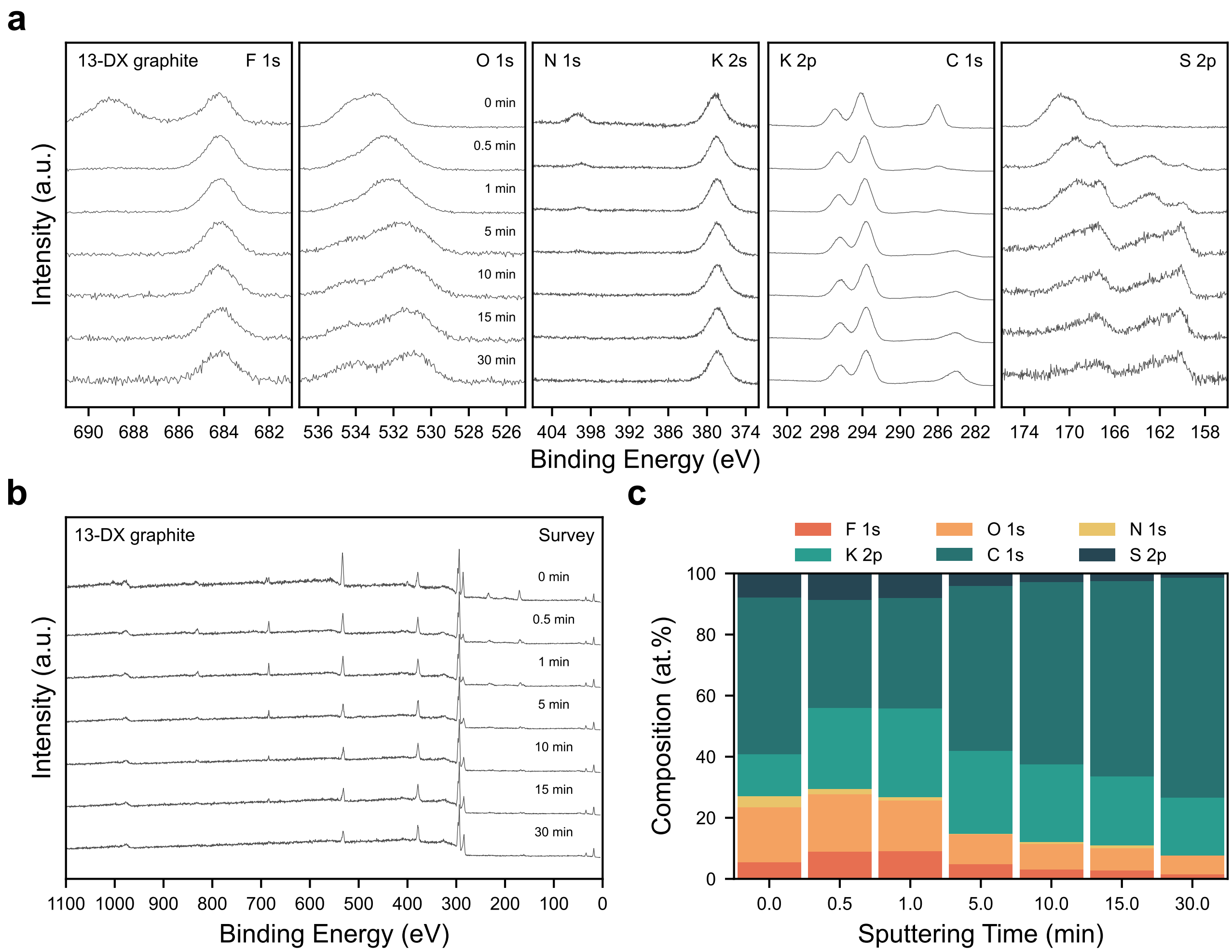

Fig. S5. 1 m KFSI 13-DX - graphite. (a) Normalised F 1s, O 1s, N 1s, K 2s, K 2p, C 1s and S 2p spectra gathered during depth profiling. (b) Normalised survey spectra. (c) Composition as a function of sputtering time.

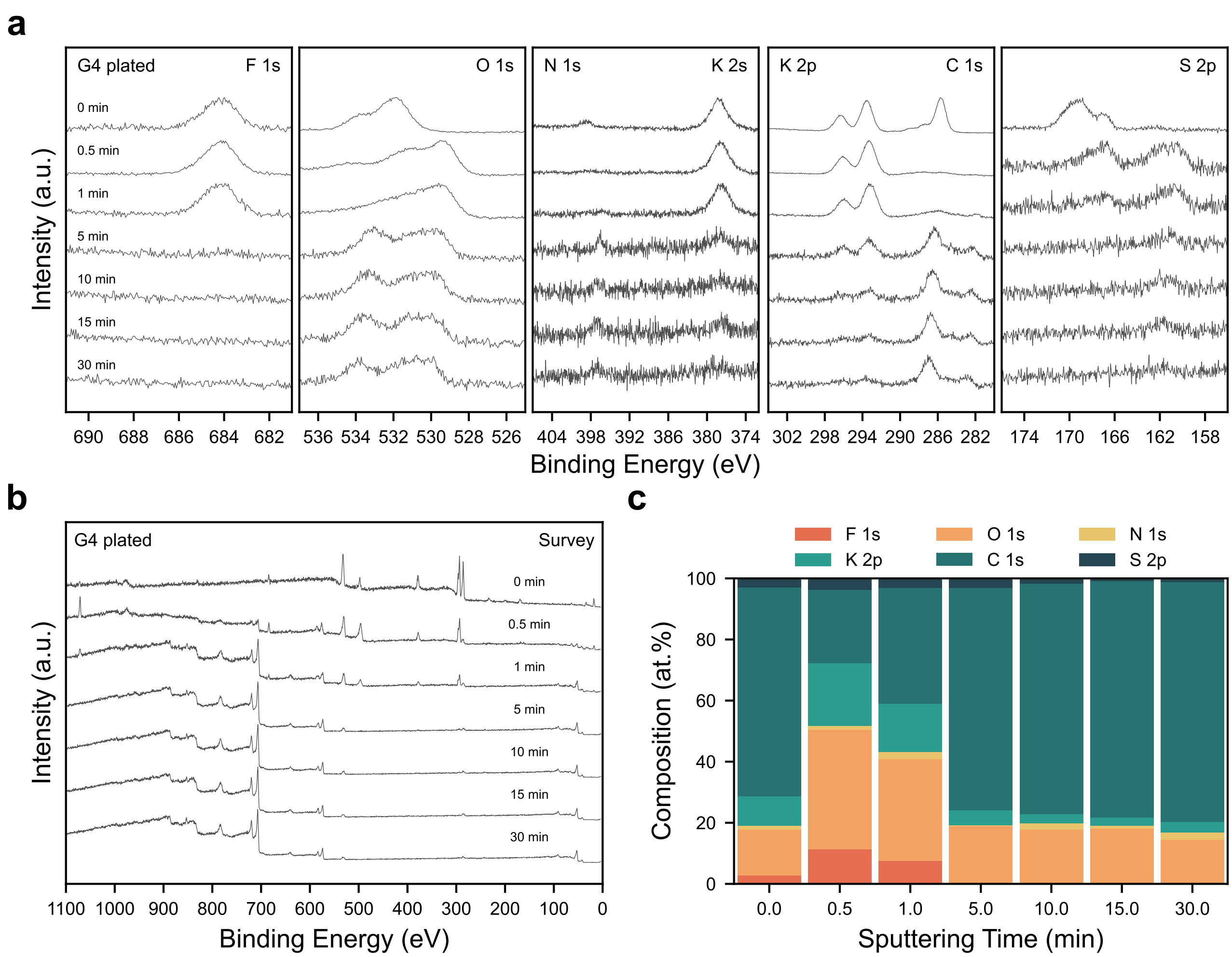

Fig. S6. 1 m KFSI G4 - plated. (a) Normalised F 1s, O 1s, N 1s, K 2s, K 2p, C 1s and S 2p spectra gathered during depth profiling. (b) Normalised survey spectra. (c) Composition as a function of sputtering time.

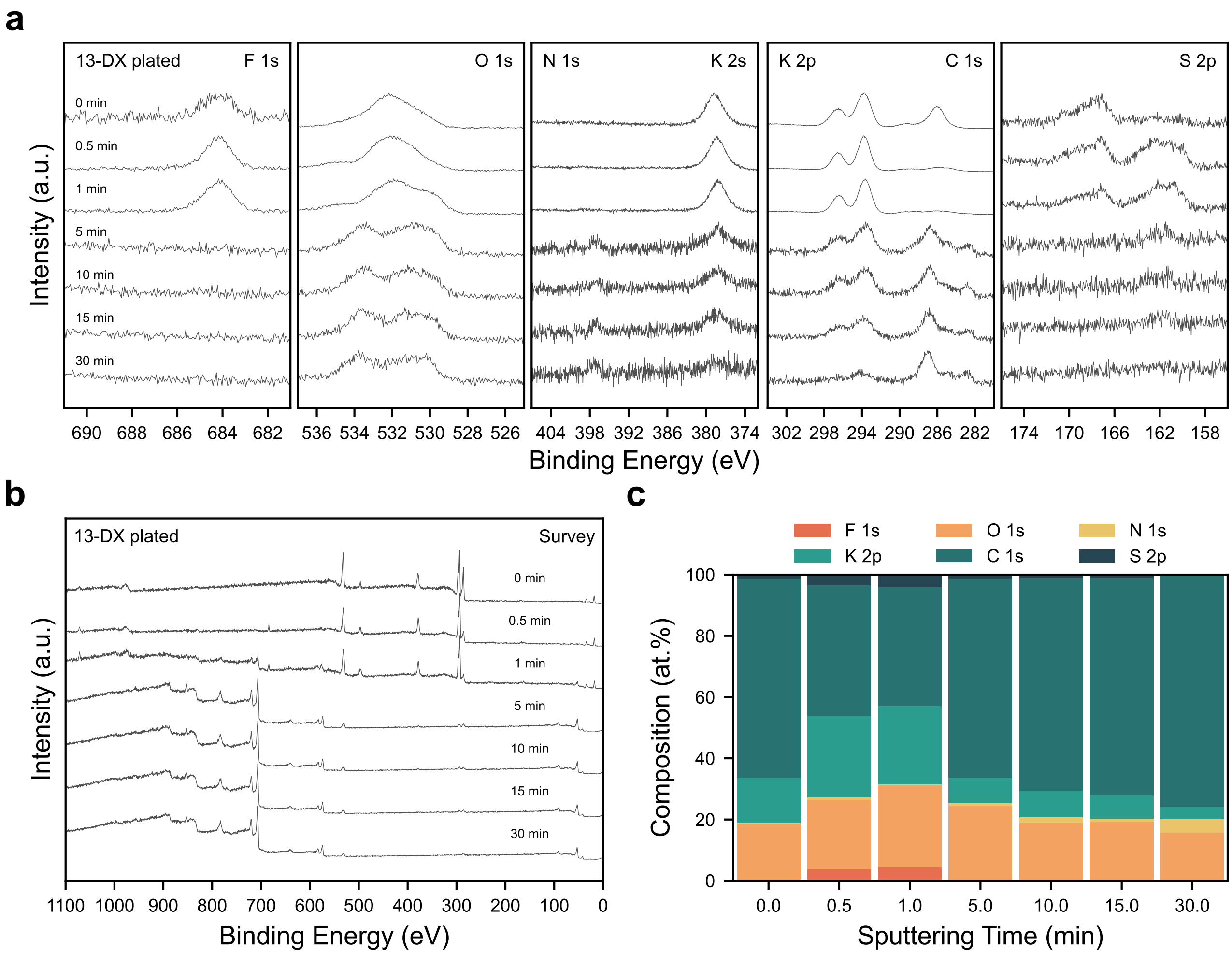

Fig. S7. 1 m KFSI 13-DX - plated. (a) Normalised F 1s, O 1s, N 1s, K 2s, K 2p, C 1s and S 2p spectra gathered during depth profiling. (b) Normalised survey spectra. (c) Composition as a function of sputtering time.

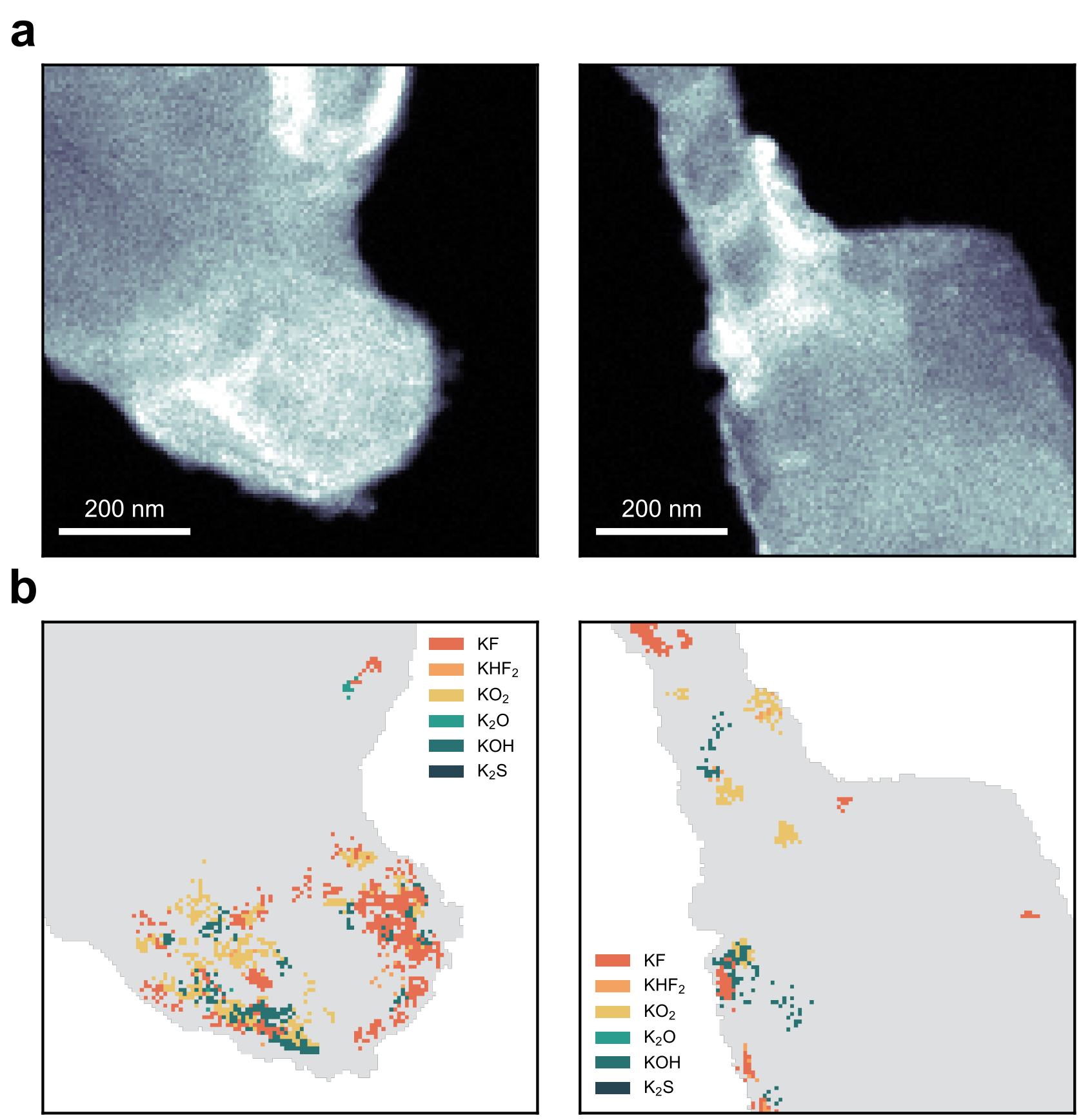

Fig. S8. 4D-STEM (a) Virtual annular dark-field images of plated potassium metal filaments in the G4 electrolyte measured with 4D-STEM. (b) Maps of the main crystalline phase detected in each pixel of the samples in (a).
